# Disability Accommodation Access and Requests in US Internal Medicine Residents With Disabilities

**DOI:** 10.1001/jamanetworkopen.2026.3392

**Published:** 2026-03-30

**Authors:** Christopher J. Moreland, Karina Pereira-Lima, Kathleen T. Lee, Elizabeth M. Viglianti, Ian A. DeAndrea-Lazarus, Andrew Zeveney, Lisa M. Meeks

**Affiliations:** 1 Department of Medicine, Dell Medical School at the University of Texas at Austin, Austin; 2 Department of Anesthesiology, University of Michigan, Ann Arbor; 3 Department of Internal Medicine, Division of Pulmonary and Critical Care, University of Michigan, Ann Arbor; 4 American College of Physicians, Philadelphia, Pennsylvania; 5 Department of Medical Education, University of Illinois College of Medicine, Chicago; 6 Department of Family Medicine, University of Michigan School of Medicine, Ann Arbor

## Abstract

**Importance:**

Despite the growing number of residents with disabilities, barriers to equitable access persist in medical training. Program access to accommodation has been linked to improved training and mental health outcomes, but little is known about possible resident and program characteristics associated with access to and requests for needed accommodations.

**Objective:**

To examine demographic, training, and disability-related factors associated with program access and accommodation requests among internal medicine (IM) residents with disabilities.

**Design, Setting, and Participants:**

This national cross-sectional study looked at accredited IM residency programs in mainland US and Puerto Rico. Participants were IM residents who took the 2023 Internal Medicine In-Training Examination and reported having at least 1 type of disability.

**Main Outcomes and Measures:**

The primary outcomes were program access, defined as receiving accommodations or not needing them, and requesting needed accommodations. Multivariable logistic regression models were conducted for each of the outcomes.

**Results:**

Of 19 205 respondents, 1824 (9.5%) reported a disability; participants were predominantly men (979 men [53.7%]), US medical graduates (1398 participants [76.6%]), and enrolled in categorical IM programs (1532 participants [84.0%]). With regard to race, 340 participants (18.6%) were Asian, 415 (22.8%) were from groups underrepresented in medicine (including self-reported Black or African American or Afro-Caribbean; Latinx or Latino or Hispanic; Native American or American Indian or Indigenous or Alaskan Native; Native Hawaiian or Pacific Islander), and 823 (45.1%) were White. Among 1052 with complete accommodation information, 811 (77.1%) had program access and 241 (22.9%) did not. In multivariable regression models, having cognitive disabilities (adjusted odds ratio [aOR], 0.27; 95% CI, 0.15-0.49) and identifying as women (aOR, 0.55; 95% CI, 0.40-0.75), Asian (aOR, 0.53; 95% CI, 0.34-0.82) and underrepresented racial or ethnic groups (aOR, 0.58; 95% CI, 0.38-0.87) were associated with lower odds of program access. Among 699 residents coded as needing disability accommodations with classifiable responses, 200 (28.6%) did not request them. Fear of stigma (164 respondents [82.0%]) and unclear institutional processes (60 respondents [30.0%]) were the most cited reasons for nonrequest for needed accommodations. Requesting accommodations was less likely among residents with cognitive disabilities (aOR, 0.16; 95% CI, 0.08-0.31) and who identify as women (aOR, 0.37; 95% CI, 0.25-0.54), genderqueer or nonbinary (aOR, 0.11; 95% CI, 0.02-0.68), Asian (aOR, 0.50; 95% CI, 0.30-0.85), or underrepresented in medicine (aOR, 0.60; 95% CI, 0.37-0.97).

**Conclusions and Relevance:**

These findings suggest that despite growing disability representation, substantial inequities in access to and requests for accommodations persisted for IM residents with disabilities, particularly those with cognitive disabilities and marginalized identities. Institutions should implement inclusive, transparent policies to foster psychological safety and disability inclusion.

## Key Points

**Question:**

What demographic, training, and disability factors are associated with accommodation access and requests among internal medicine residents with disabilities?

**Findings:**

In this cross-sectional study of 1824 internal medicine residents who reported disability, residents with cognitive disabilities, women, Asian-identifying individuals, and residents from underrepresented racial or ethnic groups had lower odds of either receiving accommodations or not needing them. Among 699 residents coded as needing accommodations and providing classifiable responses, 200 did not request, most commonly citing fear of stigma or unclear institutional processes.

**Meaning:**

These findings suggest that substantial inequities persist in accommodation access for residents and that institutions must implement disability inclusion policies.

## Teaser

This cross-sectional study examines the demographic, training, and disability-related factors associated with program access and accommodation requests among internal medicine residents with disabilities.

## Introduction

A growing number of medical students with disabilities are successfully graduating and matching into residency programs.^1,2^ As these learners enter graduate medical education (GME), it is essential that they are provided equitable access, which may include appropriate accommodations.^3^ However, despite increased disability inclusion efforts in undergraduate medical education, many residents with disabilities (RWD) encounter substantial barriers in the GME environment.^4^ These include a lack of formal disability policies,^5^ unclear processes for requesting accommodations,^6^ and limited institutional expertise to evaluate and implement reasonable accommodations.^7^

Disability-related challenges are often rooted in insufficient disability education among residency program leaders and the absence of standardized guidance on supporting RWD.^5,6,8,9^ As a result, program directors may not view accessibility as a fundamental component of medical training, but as an optional enhancement, leading to inconsistent or inadequate support across programs.

This lack of disability-related education, along with limited institutional and program-level infrastructure, has tangible consequences. Trainees with disabilities report feeling a lack of belonging, isolated, unsupported, or even experiencing discrimination.^3,10,11,12,13,14^ Program access—defined as either receiving accommodations or not needing them because the learning environment is inherently accessible—has emerged as a critical variable for predicting trainee success.^15^ Barriers to program access, such as fear of stigma, unclear institutional policies, or burdensome documentation requirements, persist.^6,8^ Prior research demonstrates that residents reporting lack of program access are more likely to experience increased depressive symptoms and self-reported medical errors compared with those with program access.^16^ Taken together, these findings underscore the connections among program access, supportive training environments, learner well-being, and patient safety.

Although research on disability and program access is growing, key gaps remain. Studies to date include small samples of RWD, limiting exploration of how program access and accommodation request patterns vary by demographic factors or program characteristics.^6,16^ As a result, we have an incomplete understanding of how RWD—particularly those with intersecting identities, such as racial or gender minoritized individuals—navigate disclosure and request accommodations. Closing these gaps is essential to developing inclusive, equitable policies that support all RWD.

To address this need, we analyzed a large, nationally representative dataset of mainland US and Puerto Rico internal medicine (IM) residents who took the 2023 American College of Physicians Internal Medicine In-Training Examination (IM-ITE). This study examines how demographic, training, and disability-related factors are associated with program access and accommodation requests among RWD.

## Methods

This cross-sectional study was approved by the University of Texas at Austin institutional review board as exempt. It follows the Strengthening the Reporting of Observational Studies in Epidemiology (STROBE) reporting guidelines.

Web-based surveys were administered to all mainland US and Puerto Rico IM resident physicians following completion of the August 2023 IM-ITE—an examination and survey administered in standard fashion across IM residency programs. Participants provided consent to have their data included in research. Only participants who completed the entire survey are included in this study. Data included self-reported disability status, receipt of accommodations from residents’ institutions, reasons for not receiving accommodations (eg, request was denied, nonrequest due to stigma, or unclear policies and procedures), resident self-reported personal and training demographics (race, ethnicity, gender, native language, program year, international medical graduate status, and type of program). The authors (C.J.M., K.P-L, and L.M.M.) previously developed the disability and accommodation questions for use in other nationally representative surveys and national data collection on training and practicing physicians^1,6,15,16,17,18^; by partnering with the American College of Physicians research leadership, these items were added to the 2023 IM-ITE resident survey (eMethods in Supplement 1).

Consistent with previous methodology, participants were included in this study if they reported yes or “I don’t know” to having a disability and reported at least 1 type of disability (eg, cognitive, motor and/or sensory, or chronic health).^6^ For participants who reported no to having a disability, the survey skipped all subsequent disability questions described in this study. We investigated the demographic, training, and disability characteristics associated with 2 primary outcomes: (1) receiving program access and (2) requesting accommodations.

### Outcome 1: Receiving Program Access

Having program access was defined as participants who reported receiving accommodations from their institution or indicated that they did not request accommodations due to not needing them. Participants who replied, “I prefer not to disclose,” to the question on whether their residency program provided accommodations for their disability were considered missing for this outcome.

### Outcome 2: Requesting Needed Accommodations

Participants were determined to have requested accommodations if they reported having received accommodations, had a request for accommodations denied, or had a request for accommodations under review. If the prior condition was not met, participants were considered as not requesting accommodations if they reported not requesting accommodations due to fear or stigma, did not have the documentation for a request, or the institution did not have a clear process for requesting accommodations. If participants selected other as the sole reason for not receiving accommodations, the write-in responses were evaluated and categorized according to whether they explicitly referred to requesting or not requesting accommodations. Participants who selected unsure as the sole reason for nonreceipt of accommodations, or who selected other without providing additional explanation, were considered missing for this outcome. Participants reporting not needing accommodations were excluded.

### Statistical Analysis

We reported the percentages and counts of the participant demographics, program training, and disability-related characteristics for participants who reported at least 1 type of disability and for those who did not. To compare demographic, training, and disability-related characteristics between groups—including participants who reported a disability vs residents who did not, participants who provided accommodation information vs those who did not, and participants who reported needing vs not needing accommodations—we used χ^2^ tests with Monte Carlo simulation (2000 replicates) to compute *P* values when cell counts were small. For multicategory variables with significant overall χ^2^ tests, standardized residuals were examined to identify which specific categories were overrepresented or underrepresented.

To evaluate factors associated with (1) receiving program access and (2) requesting needed accommodations among included participants, we conducted multivariable logistic regression models for each of the outcomes and included the following variables of interest: cognitive disability (yes or no), motor and/or sensory disability (yes or no), chronic health disability (yes or no), participant gender (men, women, genderqueer, nonbinary, or third-gender), race and/or ethnicity (Asian, Asian American, or Pan Asian; underrepresented in medicine; White; or other), native language (native English speaker or non–native English speaker), international medical graduate status (international medical graduate; US medical graduate), type of residency program (IM, categorical; IM, preliminary; IM, primary care; IM, pediatrics; or other), and postgraduate year (PGY-1, PGY-2, or PGY-3). For any free-text responses to other disability type, 2 authors with disability expertise (C.J.M. and L.M.M.) recoded these by review and consensus into existing categories (ie, cognitive, chronic health, and motor and/or sensory disability).

For race and ethnicity, we applied the Association of American Medical Colleges’ definition of underrepresented in medicine (URiM) to our study categorization, defined as “racial and ethnic populations that are underrepresented in the medical profession relative to their numbers in the general population,”^19^ which in our study corresponded to self-identifying as 1 or more of the following: American Indian or Alaska Native, Black or African American, Hispanic or Latino, or Native Hawaiian or Pacific Islander. Among our participants, the other category included Middle East and North African, or non-URiM multiracial identities (eg, Middle East and North African and/or White or White and/or Asian).

As a sensitivity analysis, we reran the program access model excluding participants who reported multiple disability categories to assess the robustness of our findings. Additionally, we conducted a sensitivity analysis restricting the program access model to only those participants classified as needing accommodations (ie, excluding those who reported not requesting accommodation because they did not need them). Statistical analysis was conducted using Stata MP version 17 (StataCorp LLC) and R statistical software version 4.4.2 (R Project for Statistical Computing), with a 2-sided *P* < .05 deemed significant.

## Results

Of 30 927 US IM resident physicians who completed the 2023 IM-ITE, 19 205 (62.10%) completed the survey. Of those, 1561 respondents (8.1%) replied yes, 15 988 (83.2%) replied no, 850 (4.4%) replied “I don’t know,” and 806 (4.2%) replied prefer not to say to the question, “are you a person with a disability?” Collectively, 1824 participants (9.5%) who replied yes or “I don’t know” went on to report at least 1 type of disability and were included in the present study. These participants were predominantly men (979 respondents [53.7%]), native English speakers (1458 respondents [79.9%]), US medical graduates (1398 respondents [76.6%]), and enrolled in categorical IM residency programs (1532 respondents [84.0%]). With regard to race, 340 participants (18.6%) were Asian, 415 (22.8%) were from URiM groups, and 823 (45.1%) were White. The most commonly reported disability category was cognitive (1397 respondents [76.6%]), followed by chronic health (230 respondents [12.6%]) and motor or sensory disabilities (159 respondents [8.7%]) (Table 1). Compared with residents who did not report a disability, included participants were less likely to be Asian (6169 participants [35.5%] vs 340 participants [18.6%]) and in categorical IM programs (15 575 participants [89.6%] vs 1532 participants [84.0%]) as well as more likely to be White (3051 participants [17.5%] vs 823 participants [45.1%]), URiM (5606 participants [32.2%] vs 415 participants [22.8%]), US medical graduates (11 119 participants [64.0%] vs 1398 participants [76.6%]), native English speakers (11 833 participants [68.1%] vs 1458 participants [79.9%]), genderqueer or nonbinary (23 participants [0.1%] vs 22 participants [1.2%]), and in primary care (806 participants [4.6%] vs 111 participants [6.1%]) or pediatrics (689 participants [4.0%] vs 124 participants [6.8%]) programs (Table 1).

**Table 1.** Demographic, Training, and Disability Characteristics of Participants

Characteristic	Participants, No. (%) (N = 19 205)	
	Reported disability (n = 1824)	Did not report disability (n = 17 381)
Demographic characteristics		
Gender		
Men	979 (53.7)	9198 (52.9)
Women	808 (44.3)	8078 (46.5)
Genderqueer, nonbinary, or third gender	22 (1.2)	23 (0.1)
Missing^a^	15 (0.9)	79 (0.5)
Race and ethnicity		
Asian, Asian American, or Pan Asian	340 (18.6)	6169 (35.5)
Underrepresented in medicine^b^	415 (22.8)	5605 (32.2)
White	823 (45.1)	3051 (17.5)
Other^c^	168 (9.2)	1771 (10.2)
Missing^a^	78 (4.3)	785 (4.5)
Native language		
English	1458 (79.9)	11 833 (68.1)
Non-English	364 (20)	5543 (31.9)
Missing^a^	2 (0.1)	5 (0.0)
Training characteristics		
International medical graduate status		
International medical graduate	425 (23.3)	6260 (36.0)
US medical graduate	1398 (76.6)	11 119 (64.0)
Missing^a^	1 (0.1)	2 (0.0)
Type of residency program		
Internal medicine, categorical	1532 (84)	15 575 (89.6)
Internal medicine, preliminary	25 (1.4)	186 (1.1)
Internal medicine, primary care	111 (6.1)	801 (4.6)
Internal medicine, pediatrics	124 (6.8)	689 (4.0)
Other	31 (1.7)	128 (0.7)
Missing^a^	1 (0.1)	2 (0.0)
Postgraduate year		
1	604 (33.1)	5636 (32.4)
2	652 (35.7)	6083 (35.0)
3	568 (31.1)	5662 (32.6)
Disability-related characteristics^d^		
Any cognitive disability	1397 (76.6)	NA
Attention-deficit/hyperactivity disorder	1245 (68.3)	NA
Learning disability	112 (6.1)	NA
Psychological disability	158 (8.7)	NA
Other cognitive disability^e^	18 (1)	NA
Any chronic health disability	230 (12.6)	NA
Chronic health disability	203 (11.1)	NA
Other chronic health disability^f^	33 (1.8)	NA
Any motor and/or sensory disability	159 (8.7)	NA
Deaf or hard of hearing	65 (3.6)	NA
Mobility disability	32 (1.8)	NA
Visual disability	60 (3.3)	NA
Other motor and/or sensory disability^g^	5 (0.3)	NA
Missing^a^	147 (8.1)	NA

Abbreviation: NA, not applicable.

^a^ Includes participants who reported prefer not to disclose or had missing values.

^b^ Underrepresented in medicine includes participants who self-reported at least 1 of the following race or ethnic identities: Latinx, Latino, or Hispanic; Native American or American Indian or Indigenous or Alaskan Native; Black or African American or Afro-Caribbean; Native Hawaiian or Pacific Islander.

^c^ Other includes participants who self-reported as Middle East and North African, or non-URiM multiracial (eg, Middle East and North African and White, White and Asian, or Middle East and North African and Asian).

^d^ Participants were allowed to select more than 1 type of disability. Percentages may sum to greater than 100%. A total of 94 participants reported disabilities spanning multiple categories.

^e^ Other cognitive disability was coded based on participants’ open-text responses and includes autism spectrum, depression, Tourette syndrome, generalized anxiety disorder, delayed processing, and obsessive-compulsive disorder.

^f^ Other chronic health disability was coded based on participants’ open-text responses and includes rheumatologic disorder, autoimmune disorders, diabetes, chronic migraines, chronic pain, narcolepsy, severe hypertension, and seizure disorders.

^g^ Other motor and/or sensory disability was coded based on participants’ open-text responses and includes limb amputation, genetic movement disorder, amniotic band syndrome, and ankylosing spondylitis.

### Program Access to Accommodations

Among the 1824 eligible participants, 1052 (57.7%) provided information on whether their program offered disability accommodations and reasons for not receiving accommodations in case those were not received. Compared with residents who did not provide information on program accommodations, they were more likely to be White (303 participants [39.2%] vs 520 participants [49.4%]) and native English speakers (593 participants [76.8%] vs 865 participants [82.2%]) and to report any chronic health (65 participants [8.4%] vs 165 participants [15.7%]) or any motor and/or sensory (51 participants [6.6%] vs 165 participants [10.3%]) disability, and less likely to be Asian (170 participants [22.0%] vs 170 participants [16.2%]), from a racial or ethnic group classified as other (84 participants [10.9%] vs 84 participants [8.0%]), or an international medical graduate (211 participants [27.3%] vs 214 participants [20.3%]) (eTable 1 in Supplement 1). Among those who provided information on accommodations, 811 participants (77.1%) were classified as having program access, and 241 (22.9%) were classified as lacking program access. Of the 811 RWD with program access, 482 (59.4%) reported receiving accommodations through their program and 329 RWD (40.6%) stated they did not request accommodations because they did not need them. Among the 241 RWD lacking program access, the majority (200 [83.0%]) had not requested needed accommodations, while 10 RWD (4.1%) reported having their accommodation requests denied, 7 (2.9%) had requests under review, and 24 (10.0%) reported being unsure about the reason for not receiving accommodations from their program.

In a multivariable logistic regression model adjusting for participant demographics, training, and disability characteristics, several factors were associated with lower odds of having program access to accommodations. These included having a cognitive disability (adjusted odds ratio [aOR], 0.27; 95% CI, 0.15-0.49) compared with not having a cognitive disability, being a woman (aOR, 0.55; 95% CI, 0.40-0.75) compared with being a man, and identifying as Asian (aOR, 0.53; 95% CI, 0.34-0.82) or belonging to a race or ethnicity URiM (aOR, 0.58; 95% CI, 0.38-0.87) compared with residents identifying as White (Table 2). Sensitivity analysis excluding individuals reporting multiple disability categories did not significantly alter these results (eTable 2 in Supplement 1).

**Table 2.** Multivariable Logistic Regression Assessing Characteristics Associated With Receiving Program Access

Characteristic	aOR (95% CI)	*P* value
Disability-related characteristics		
No	1 [Reference]	NA
Any cognitive disability	0.27 (0.15-0.49)	<.001
Any chronic health disability	0.81 (0.46-1.42)	.47
Any motor and/or sensory disability	0.98 (0.48-2.01)	.97
Demographic characteristics		
Gender		
Men	1 [Reference]	NA
Women	0.55 (0.40-0.75)	<.001
Genderqueer, nonbinary, or third gender	0.34 (0.10-1.13)	.08
Race or ethnicity		
Asian	0.53 (0.34-0.82)	.005
Underrepresented in medicine^a^	0.58 (0.38-0.87)	.009
White	1 [Reference]	NA
Other^b^	0.68 (0.39-1.21)	.20
Native language		
English	1 [Reference]	NA
Non-English	1.16 (0.73-1.85)	.54
Training characteristics		
Medical graduate status		
US medical graduate	1 [Reference]	NA
Internal medical graduate	0.78 (0.52-1.17)	.24
Type of residency program		
Internal medicine [categorical]	1 [Reference]	NA
Internal medicine (preliminary)	0.50 (0.14-1.86)	.30
Internal medicine - primary care	0.95 (0.52-1.76)	.88
Internal medicine - pediatrics	1.55 (0.82-2.94)	.18
Other	2.28 (0.50-10.28)	.28
Postgraduate year		
1	1 [Reference]	NA
2	0.82 (0.56-1.21)	.32
3	0.72 (0.49-1.06)	.10

Abbreviations: aOR, adjusted odds ratio; NA, not applicable.

^a^ Underrepresented in medicine includes participants who self-reported at least one of the following racial or ethnic identities: Latinx, Latino, or Hispanic; Native American, American Indian, Indigenous or Alaskan Native; Black, African American, Afro-Caribbean; Native Hawaiian, Pacific Islander.

^b^ Other includes participants who self-reported as Middle East and North African, or non-underrepresented in medicine multiracial (eg, Middle East and North African and/or White, White and Asian, or Middle East and North African and/or Asian).

### Program Access Among Residents With Need for Accommodation

Compared with 329 residents who reported not needing disability accommodations, 723 residents whose responses were classified as needing disability accommodations were more likely to be from URiM groups (57 participants [17.3%] vs 180 participants [24.9%]), to be international medical graduates (52 participants [15.8%] vs 162 participants [22.4%]), and to report a chronic health (28 participants [8.5%] vs 137 participants [18.9%]) or motor and/or sensory (17 participants [5.2%] vs 91 participants [12.6%]) disability, and less likely to be White (182 participants [55.3%] vs 338 participants [46.7%]) or to report a cognitive disability (288 participants [87.5%] vs 512 participants [70.8%]) (eTable 3 in Supplement 1). The multivariable logistic regression model for program access restricted to participants classified as needing accommodations yielded results similar to those of the model including all participants, with the following factors associated with lower odds of receiving program access: having a cognitive disability (aOR, 0.22; 95% CI, 0.12-0.38) compared with not having a cognitive disability, being a woman (aOR, 0.42; 95% CI, 0.29-0.60) or identifying as nonbinary or genderqueer or third-gender (aOR. 0.13; 95% CI, 0.02-0.67) compared with being a man, and identifying as Asian (aOR, 0.54; 95% CI, 0.33-0.89) or belonging to a race and ethnicity URiM (aOR, 0.63; 95% CI, 0.40-0.99) compared with residents identifying as White (Table 3).

**Table 3.** Multivariable Logistic Regression Assessing Characteristics Associated With Receiving Program Access Among Participants Classified as Needing Accommodations

Characteristic	aOR (95% CI)	*P* value
Disability-related characteristics		
No disability	1 [Reference]	NA
Any cognitive disability	0.22 (0.12-0.38)	<.001
Any chronic health disability	1.22 (0.69-2.20)	.50
Any motor and/or sensory disability	1.45 (0.71-3.09)	.30
Demographic characteristics		
Gender		
Men	1 [Reference]	NA
Women	0.42 (0.29-0.60)	<.001
Genderqueer, nonbinary, third gender	0.13 (0.02-0.67)	.02
Race or ethnicity		
Asian	0.54 (0.33-0.89)	.02
Underrepresented in medicine^a^	0.63 (0.40-0.99)	.046
White	1 [Reference]	NA
Other^b^	0.61 (0.31-1.19)	.14
Native language		
English	1 [Reference]	NA
Non-English	1.27 (0.75-2.14)	.40
Training characteristics		
Medical graduate status		
US medical graduate	1 [Reference]	NA
Internal medical graduate	0.89 (0.56-1.43)	.60
Type of residency program		
Internal medicine, categorical	1 [Reference]	NA
Internal medicine, preliminary	0.50 (0.11-2.32)	.40
Internal medicine, primary care	1.22 (0.63-2.42)	.60
Internal medicine, pediatrics	1.64 (0.82-3.46)	.20
Other	2.71 (0.63-18.7)	.20
Postgraduate year		
1	1 [Reference]	NA
2	0.77 (0.50-1.19)	.20
3	0.68 (0.44-1.05)	.08

Abbreviations: aOR, adjusted odds ratio; NA, not applicable.

^a^ Underrepresented in medicine includes participants who self-reported at least 1 of the following racial or ethnic identities: Latinx, Latino, Hispanic; Native American, American Indian or Indigenous or Alaskan Native; Black or African American or Afro-Caribbean; Native Hawaiian or Pacific Islander.

^b^ Other includes participants who self-reported as Middle East and North African (MENA), or non-underrepresented in medicine multiracial (eg, MENA and/or White, White and/or Asian, MENA and/or Asian).

### Reasons for Not Requesting Needed Accommodations

Among 723 RWD with reported need for disability accommodations, 699 provided responses that indicated whether they had requested accommodations; 24 were excluded for selecting only unsure (23 RWD) or other without additional explanation (1 RWD). Of those 699 RWD, 499 (71.4%) requested accommodations, of whom 482 (96.6%) had accommodations provided, 10 (2.0%) had their accommodation request denied, and 7 (1.4%) reported their accommodation request was under review. Among the 200 RWD (28.6%) who did not request needed accommodations, the most commonly reported reasons for nonrequest were fear of stigma or bias (164 respondents [82.0%]) and the absence of a clear institutional process for requesting accommodation (60 respondents [30.0%]) (Figure).

**Figure.**
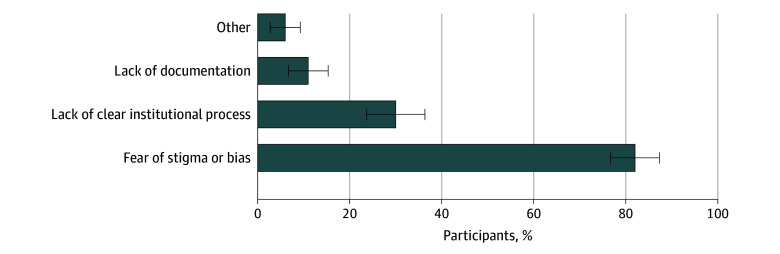
Bar Graph of Reported Reasons for Not Requesting Needed Disability Accommodations Residents were allowed to select more than 1 reason. Open responses to other reasons to not request accommodations include missed the deadline to request, did not disclose diagnosis to institution, did not look into the type of accommodations or whether accommodations are available, and believed that it would be difficult to acquire accommodations. Error bars represent 95% CIs for the percentage estimates, calculated using the normal approximation to the binomial distribution.

Multivariable analyses of participant characteristics associated with requesting accommodations when needed showed that residents with a cognitive disability (aOR, 0.16; 95% CI, 0.08-0.31) compared with those without a cognitive disability, women (aOR, 0.37; 95% CI, 0.25-0.54) and genderqueer, nonbinary, or third-gender individuals (aOR, 0.11; 95% CI, 0.02-0.68) compared with men, Asian individuals (aOR, 0.50; 95% CI, 0.30-0.85) compared with White individuals), and those from underrepresented racial or ethnic groups (aOR, 0.60; 95% CI, 0.37-0.97) compared with White individuals were significantly less likely to request accommodations when needed (Table 4).

**Table 4.** Multivariable Logistic Regression Assessing Characteristics Associated With Requesting Needed Accommodations

Characteristic	aOR (95% CI)	*P* value
Disability-related characteristics		
No disability	1 [Reference]	NA
Any cognitive disability	0.16 (0.08-0.31)	<.001
Any chronic health disability	1.16 (0.63-2.15)	.63
Any motor and/or sensory disability	0.97 (0.46-2.07)	.94
Demographic characteristics		
Gender		
Men	1 [Reference]	NA
Women	0.37 (0.25-0.54)	<.001
Genderqueer or nonbinary or third gender	0.11 (0.02-0.68)	.02
Race or ethnicity		
White	1 [Reference]	NA
Asian	0.50 (0.30-0.85)	.01
Underrepresented in medicine^a^	0.60 (0.37-0.97)	.04
Other^b^	0.68 (0.33-1.40)	.30
Native language		
English	1 [Reference]	NA
Non-English	1.42 (0.80-2.49)	.23
Training characteristics		
Medical graduate status		
US medical graduate	1 [Reference]	NA
Internal medical graduate	0.99 (0.59-1.64)	.96
Type of residency program		
Internal medicine, categorical	1 [Reference]	NA
Internal medicine, preliminary	0.40 (0.09-1.75)	.22
Internal medicine, primary care	1.08 (0.54-2.14)	.83
Internal medicine, pediatrics	1.55 (0.74-3.24)	.25
Other	2.17 (0.43-10.90)	.35
Postgraduate year		
1	1 [Reference]	NA
2	0.83 (0.52-1.31)	.42
3	0.79 (0.50-1.25)	.31

Abbreviations: aOR, adjusted odds ratio; NA, not applicable.

^a^ Underrepresented in medicine includes participants who self-reported at least 1 of the following racial or ethnic identities: Latinx or Latino or Hispanic; Native American or American Indian or Indigenous or Alaskan Native; Black or African American or Afro-Caribbean; Native Hawaiian or Pacific Islander.

^b^ Other includes participants who self-reported as Middle East and North African, or non-underrepresented in medicine multiracial (eg, Middle East and North African and/or White, White and/or Asian, or Middle East and North African and/or Asian).

## Discussion

To our knowledge, this cross-sectional study provides the first nationally representative analysis of disability accommodation among residents in IM, the largest specialty in graduate medical education. Drawing on data from mainland US and Puerto Rico resident physicians who participated in the 2023 IM-ITE, we found that about 1 in 4 RWD (22.9%) in our sample were classified as lacking program access. Residents with cognitive disabilities had markedly lower odds of having program access compared with those without cognitive disabilities. Access disparities were also observed among residents with different demographic characteristics: women, Asian residents, and those identifying with racial or ethnic groups URiM were significantly less likely to report program access compared with peers who were men or White. These findings highlight the need for stronger institutional support for RWD and clearer structures to ensure equitable access to accommodations across all trainees.

IM has been identified as one of the specialties where RWD applicants were significantly more likely to match compared with their nondisabled applicant peers.^2^ Our study found that 9.5% of IM residents reported at least 1 disability. Consistent with previous research on disability among medical students, attention-deficit/hyperactivity disorder and other cognitive and psychological disabilities were most commonly reported, followed by chronic health conditions and then physical and sensory disabilities.^15^

While most RWD (811 respondents [77.1%]) in our sample were coded as having program access—either through receiving accommodations or not needing them—nearly 1 in 4 RWD (22.9%) were coded as lacking program access. These findings align with prior research across multiple specialties, which suggests that key barriers to disability accommodations include fear of stigma and bias, unclear accommodation procedures, and lack of disability-related documentation. Similarly, consistent with studies among medical students with disabilities showing lower rates of program access among those with cognitive disabilities, our findings indicate that residents with cognitive disabilities had lower odds of both program access and requesting needed accommodations.^18^ Of note, 40.6% of those with program access reported not needing accommodations; this could be due to respondents’ self-adapting to optimize access or working in a residency program, clinical climate, or specialty structured such that access is more often present for those RWD without the need to request accommodations. Notably, residents who reported not needing accommodations differed meaningfully from those classified as needing them: they were more likely to be White and to report cognitive disabilities and less likely to be URiM, to be international medical graduates, or to report chronic health or motor-sensory disabilities. These differences may reflect the distinct nature of disability types. While chronic health, motor, and sensory disabilities are often more apparent and potentially require more visible accommodations (eg, physical modifications or scheduling adjustments for medical appointments), cognitive disabilities are often nonapparent and historically stigmatized in medicine, which may lead residents with these disabilities to navigate their access needs without formal accommodation requests when possible.

Leveraging the national scale of our respondent population, we examined disparities in program access across gender, race, and ethnicity. We found that RWD who identified as women, Asian, or URiM were significantly less likely to receive program access. These disparities persisted in analyses restricted to residents who reported needing accommodations and were similarly observed in analyses of whether residents requested accommodations when needed, suggesting that demographic disparities in accommodation access are robust and not attributable to differences in accommodation need. These findings raise important concerns about the role of societal and structural factors—such as stigma, cultural norms, and the intersection of ableism, sexism, and racism—in shaping disclosure and accommodation requests. Ableism in medical training, including the belief that individuals with disabilities are inherently less competent, is a known factor associated with nondisclosure.^20^ Additionally, bias may have shaped program directors’ perceptions of both the necessity of accommodations and the legitimacy of disability identities.^8^ Notably, no statistically significant differences were observed for program access or accommodation requested based on international medical graduate status or PGY, suggesting that disparities may be more closely tied to social identity than to length or type of undergraduate medical training.

Encouragingly, over 96% of RWD who requested accommodations had their requests approved, consistent with trends observed in undergraduate medical education, highlighting the importance of streamlining pathways and empowering learners to effectively submit needed accommodation requests.^18^ However, among those in our study who were classified as lacking program access, the large majority (83.0%) had not submitted a request. In line with previous studies, the 2 primary reasons cited in this study for not requesting accommodations were fear of stigma and uncertainty about program policies or procedures for disclosure; rather than institutional denial, this pattern points to a broader absence of psychological safety around disability disclosure and limited institutional transparency in how accommodation requests are processed.^6^ Importantly, our findings add to this literature demonstrating that this nonrequesting pattern was especially pronounced among residents with cognitive disabilities and among those who identified as women; genderqueer, nonbinary, or third gender; Asian; or from racial or ethnic groups historically URiM.

Our findings identify several areas for improvement toward disability-inclusive learning and working climates. In alignment with previous recommendations and the Accreditation Council for Graduate Medical Education (ACGME) requirements,^21^ residency program leadership and sponsoring institutions must develop clear, publicly accessible policies and procedures that specify to whom disability disclosure and accommodation requests should be directed, how those requests will be evaluated, and how the effectiveness of accommodations will be continuously assessed.^9^ Programs and clinical partners—including and not limited to hospitals and clinics—should also implement antiableist training for all faculty and leadership. Such training should cover disability inclusion, the benefits of a diverse workforce, and practical strategies for fostering psychologically safe environments that support disclosure without fear of stigma or reprisal. Although clear guidelines exist to support residency programs in developing such policies, their implementation remains uneven. For example, research has shown that clear institutional policies and dedicated support from disability resource professionals can facilitate more equitable accommodation processes, yet many residency programs still lack standardized procedures.^5,22,23^ Importantly, given our findings on the intersection of disability, gender, and race and ethnicity, such training and protective workforce policies should also address the compounded barriers faced by RWD from diverse demographic identities.

Notably, our data suggest that hesitancy to disclose disability status may be widespread. Over 4% of respondents preferred not to indicate whether they had a disability, despite “I don’t know” and no- being available options. This pattern raises questions about whether respondents felt safe in reporting such information in their GME environment. Even more striking, 42% of residents who endorsed having a disability chose not to indicate whether their programs had provided accommodations. Even though respondents were informed that none of their survey responses would be shared with their program, some residents may have had concerns that their disclosures could be traced back to them. Similar concerns have been explicitly described by medical students in qualitative responses to national surveys.^24^ Importantly, residents who did not provide accommodation information differed systematically from those who did: they were more likely to be Asian, international medical graduates, or from racial and ethnic groups classified as other and less likely to be White, to be native English speakers, or to report chronic health or motor and/or sensory disabilities. This pattern suggests that hesitancy to disclose may be particularly pronounced among residents from international or nondominant cultural backgrounds, potentially reflecting different cultural norms around disability disclosure, concerns about employment or immigration implications, or compounded experiences of marginalization. Such hesitancy may reflect not only a lack of trust in local program environments but also fears of downstream career consequences. As of 2022, only 3 of the 55 US states and territorial medical licensing boards had fully implemented the Federation of State Medical Boards recommendations for compliance with the Americans with Disabilities Act and promotion of physician wellness.^25^ While other reasons for nonresponse may exist, this broader policy context may further discourage disclosure, even in training and research settings where confidentiality is emphasized.

### Limitations

This study has limitations. The survey did not capture whether residents’ disabilities were present before or developed during residency, which may play a role in a resident’s knowledge of their disability identity, access needs, and experience in communicating those needs effectively to proper program and institutional representatives. This survey also does not include information about the presence or use of specialized disability resource professionals.^22^ Additionally, the quality of accommodations granted, from the perspective of both RWD and program directors, could not be assessed here. Another limitation in interpreting our findings is the apparent hesitancy or discomfort some residents had when responding to disability and accommodation questions, as suggested by the high rate of nonresponse or ambiguous responses to these items. Supplementary comparisons of residents who provided vs did not provide accommodation information, and those who needed vs did not need accommodations, revealed systematic differences by race and ethnicity, international medical graduate status, and disability type. While these comparisons offer insights into potential barriers to disclosure, they also suggest that nonresponse was not random; had these residents responded, our estimates of program access and accommodation requests could have differed. Furthermore, while 62% of residents who completed the IM-ITE also completed the voluntary survey, we cannot fully assess whether survey completers differ from noncompleters. The self-reported nature of this survey, while not inclusive of other disability-relevant perspectives such as diagnostic documentation, is consistent with the extensive disability research and reflective of the lived disability experience.

## Conclusions

Despite nearly 1 in 10 residents reporting a disability, requests for and access to accommodations were markedly lower among women, Asian residents, those from groups underrepresented in medicine, and residents with cognitive disabilities. Future research should pursue similar data collection across other specialties, providing valuable interspecialty comparisons. Residency programs can take steps to improve psychological safety for RWD by engaging with the available resources for disability inclusion in GME, including the Docs with Disabilities Initiative Disability in Graduate Medical Education community of practice website and listserv,^26^ and specific ACGME requirements on disability inclusion.^21^ Finally, linking longitudinal data from pre–medical education through residency and subsequent practice would offer valuable opportunities for organizations and accrediting bodies to identify patterns and pinpoint areas of vulnerability across the clinical training and practice continuum. Such insights could illuminate critical transition points—from entry into medical school to the shift into residency and beyond—as learners become independently practicing physicians.

## Notes

Supplement 1. eMethods. Disability Questions Utilized in the Present Study eTable 1. Characteristics of Participants Who Provided vs. Did Not Provide Information on Program Accommodations (N = 1824) eTable 2. Sensitivity Analysis Assessing Characteristics Associated with Receiving Program Access, Excluding Participants with Multiple Disability Categories (N = 945) eTable 3. Characteristics of Participants Classified as Needing vs. Not Needing Disability Accommodations (N = 1052)

Supplement 2. Data Sharing Statement
