# Supplementary material for: Disability Accommodation Access and Requests in US Internal Medicine Residents With Disabilities
Source: JAMA Netw Open. 2026 Mar 30;9(3):e263392. doi: 10.1001/jamanetworkopen.2026.3392 (PMC13036577; doi:10.1001/jamanetworkopen.2026.3392)
Supplement: Supplement 1. — eMethods. Disability Questions Utilized in the Present Study eTable 1. Characteristics of Participants Who Provided vs. Did Not Provide Information on Program Accommodations (N = 1824) eTable 2. Sensitivity Analysis Assessing Characteristics Associated with Receiving Program Access, Excluding Participants with Multiple Disability Categories (N = 945) eTable 3. Characteristics of Participants Classified as Needing vs. Not Needing Disability Accommodations (N = 1052) [file jamanetwopen-e263392-s001.pdf]

Supplemental Online Content

Moreland CJ, Pereira-Lima K, Lee KT, et al. Disability accommodation access and requests in US internal medicine residents with disabilities. *JAMA Netw Open.* 2026;9(3):e263392. doi:10.1001/jamanetworkopen.2026.3392

eMethods. Disability Questions Utilized in the Present Study

eTable 1. Characteristics of Participants Who Provided vs. Did Not Provide Information on Program Accommodations (N = 1824)

eTable 2. Sensitivity Analysis Assessing Characteristics Associated with Receiving Program Access, Excluding Participants with Multiple Disability Categories (N = 945)

eTable 3. Characteristics of Participants Classified as Needing vs. Not Needing Disability Accommodations (N = 1052)

This supplemental material has been provided by the authors to give readers additional information about their work.

© 2026 Moreland CJ et al. *JAMA Network Open*

eMethods. Disability questions utilized in the present study

1) Are you a person with a disability (e.g., ADHD, learning, psychological, chronic health, mobility, hearing, vision)?
    a. Yes
    b. No [ENDS SURVEY]
    c. I do not know
    d. Prefer not to say [ENDS SURVEY]

2) Which of the following best describes your disability? If you have more than one type, select all that apply.
    a. Attention deficit/hyperactivity disorder
    b. Chronic health disability
    c. Deaf or hard of hearing
    d. Learning disability
    e. Mobility disability
    f. Psychological disability
    g. Visual disability
    h. Other: _______

3) Has your residency program provided accommodations for your disability?
    a. Yes [ENDS SURVEY]
    b. No
    c. Prefer not to say [ENDS SURVEY]

4) Which of the following best describes why your residency program did not or has not provided accommodations? Choose all that apply.
    a. My request for accommodations was denied
    b. My request for accommodations is under review
    c. I have not requested accommodations because I feel I do not need accommodations
    d. I have not requested accommodations due to fear of stigma or bias
    e. I do not have documentation to support my request
    f. My institution does not have a clear process for requesting accommodation
    g. Other _____
    h. Unsure

© 2026 Moreland CJ et al. *JAMA Network Open*

eTable 1. Characteristics of Participants Who Provided vs. Did Not Provide Information on Program Accommodations (N = 1824)

**Characteristics**	**Did not Provide Information (N = 772)**	**Provided Information (N=1,052)**
Demographic characteristics, Frequency (%)		
Gender		
Men	417 (54.0%)	562 (53.4%)
Women	337 (43.7%)	471 (44.8%)
Genderqueer/Non-binary/third gender	9 (1.2%)	13 (1.2%)
Missing [a]	9 (1.2%)	6 (0.6%)
Race/Ethnicity		
Asian, Asian American, or Pan Asian	170 (22.0%)	170 (16.2%)
Underrepresented in medicine [b]	178 (23.1%)	237 (22.5%)
White	303 (39.2%)	520 (49.4%)
Other [c]	84 (10.9%)	84 (8.0%)
Missing [a]	37 (4.8%)	41 (3.9%)
Native Language		
English	593 (76.8%)	865 (82.2%)
Non-English	179 (23.2%)	185 (17.6%)

© 2026 Moreland CJ et al. *JAMA Network Open*

Missing [a]	0 (0.0%)	2 (0.2%)
Training characteristics, Frequency (%)		
International medical graduate status		
International medical graduate	211 (27.3%)	214 (20.3%)
U.S. medical graduate	561 (72.7%)	837 (79.6%)
Missing [a]	0 (0.0%)	1 (0.1%)
Type of Residency Program		
Internal Medicine (categorical)	660 (85.5%)	872 (82.9%)
Internal Medicine (preliminary)	14 (1.8%)	11 (1.0%)
Internal Medicine - Primary Care	41 (5.3%)	70 (6.7%)
Internal Medicine - Pediatrics	43 (5.6%)	81 (7.7%)
Other	14 (1.8%)	17 (1.6%)
Missing [a]	0 (0.0%)	1 (0.1%)
Postgraduate year (PGY)		
PGY-1	264 (34.2%)	340 (32.3%)
PGY-2	289 (37.4%)	363 (34.5%)
PGY-3	219 (28.4%)	349 (33.2%)
Disability-related characteristics,[d] Frequency (%)		
Any cognitive disability	597 (77.3%)	800 (76.0%)

© 2026 Moreland CJ et al. *JAMA Network Open*

Any chronic health disability	65 (8.4%)	165 (15.7%)
Any motor and/or sensory disability	65 (8.4%)	165 (15.7%)
Missing [a]	51 (6.6%)	108 (10.3%)

[a]*Includes participants who reported "Prefer not to disclose" or had missing values*
[b]*Under-represented in medicine (URiM) includes participants who self-reported at least one of the following race/ethnic identities: Latinx/Latino/Hispanic; Native American/American Indian/Indigenous or Alaskan Native; Black/African American/Afro-Caribbean; Native Hawaiian/Pacific Islander.*
[c]*Other includes participants who self-reported as Middle East and North African (MENA), or non-URIM multiracial (e.g., MENA/White, White/Asian, MENA/Asian).*
[d]*Participants were allowed to select more than one type of disability. Percentages may sum to greater than 100%.*

eTable 2. Sensitivity Analysis Assessing Characteristics Associated with Receiving Program Access, Excluding Participants with Multiple Disability Categories (N = 945)

**Characteristic**	**Adjusted Odds Ratio of receiving program access (95% CI)**	**P-Value**
Disability-related characteristics		
Any cognitive disability: yes (reference: no)	**0.37 (0.14, 0.85)**	**0.031**
Any chronic health disability: yes (reference: no)	1.24 (0.40, 3.52)	0.7
Any motor and/or sensory disability: yes (reference: no)	1.65 (0.48, 5.70)	0.4
Demographic characteristics		
Gender		

Women (reference: men)	**0.56 (0.41, 0.78)**	**<0.001**
Genderqueer/Non-binary/third gender (reference: men)	0.13 (0.02, 0.67)	0.053
Race/Ethnicity		
Asian (reference: White)	**0.53 (0.34, 0.84)**	**0.006**
Underrepresented in medicine [a] (reference: White)	**0.62 (0.40, 0.96)**	**0.030**
Other [b] (reference: White)	0.64 (0.36, 1.16)	0.13
Native Language		
Non-English (reference: English)	1.18 (0.73, 1.93)	0.5
Training characteristics		
Medical graduate status		
Internal medical graduate (reference: US medical graduate)	0.75 (0.49, 1.15)	0.2
Type of Residency Program		
Internal Medicine (preliminary) (reference: Internal Medicine [categorical])	0.40 (0.11, 1.69)	0.2
Internal Medicine - Primary Care (reference: Internal Medicine [categorical])	0.87 (0.47, 1.70)	0.7
Internal Medicine - Pediatrics (reference: Internal Medicine [categorical])	1.64 (0.86. 3.43)	0.2
Other (reference: Internal Medicine [categorical])	1.90 (0.50, 12.4)	0.4
Postgraduate year (PGY)		
PGY-2 (reference: PGY-1)	0.89 (0.59, 1.33)	0.6
PGY-3 (reference: PGY-1)	0.76 (0.51, 1.13)	0.2

[a] Under-represented in medicine (URiM) includes participants who self-reported at least one of the following race/ethnic identities: Latinx/Latino/Hispanic; Native American/American Indian/Indigenous or Alaskan Native; Black/African American/Afro-Caribbean; Native Hawaiian/Pacific Islander.
[b]Other includes participants who self-reported as Middle East and North African (MENA), or non-URIM multiracial (e.g., MENA/White, White/Asian, MENA/Asian).

© 2026 Moreland CJ et al. *JAMA Network Open*

eTable 3: Characteristics of Participants Classified as Needing vs. Not Needing Disability Accommodations (N = 1052)

**Characteristics**	**Not needing disability accommodations (N = 329)**	**Needing disability accommodations (N = 723)**
Demographic characteristics, Frequency (%)		
Gender		
Men	172 (52.3%)	390 (53.9%)
Women	150 (45.6%)	321 (44.4%)
Genderqueer/Non-binary/third gender	6 (1.8%)	7 (1.0%)
Missing [a]	0 (0.0%)	1 (0.1%)
Race/Ethnicity		
Asian, Asian American, or Pan Asian	49 (14.9%)	121 (16.7%)
Underrepresented in medicine [b]	57 (17.3%)	180 (24.9%)
White	182 (55.3%)	338 (46.7%)
Other [c]	31 (9.4%)	53 (7.3%)
Missing [a]	10 (3.0%)	31 (4.3%)
Native Language		
English	287 (87.2%)	578 (79.9%)
Non-English	41 (12.5%)	144 (19.9%)
Missing [a]	1 (0.3%)	1 (0.1%)
Training characteristics, Frequency (%)		

© 2026 Moreland CJ et al. *JAMA Network Open*

International medical graduate status		
International medical graduate	52 (15.8%)	162 (22.4%)
U.S. medical graduate	277 (84.2%)	560 (77.5%)
Missing [a]	0 (0.0%)	1 (0.1%)
Type of Residency Program		
Internal Medicine (categorical)	274 (83.3%)	598 (82.7%)
Internal Medicine (preliminary)	3 (0.9%)	8 (1.1%)
Internal Medicine - Primary Care	17 (5.2%)	53 (7.3%)
Internal Medicine - Pediatrics	28 (8.5%)	53 (7.3%)
Other	7 (2.1%)	10 (1.4%)
Missing [a]	0 (0.0%)	1 (0.1%)
Postgraduate year (PGY)		
PGY-1	105 (31.9%)	235 (32.5%)
PGY-2	115 (35.0%)	248 (34.3%)
PGY-3	109 (33.1%)	240 (33.2%)
Disability-related characteristics,[d] *Frequency (%)*		
Any cognitive disability	288 (87.5%)	512 (70.8%)
Any chronic health disability	28 (8.5%)	137 (18.9%)
Any motor and/or sensory disability	17 (5.2%)	91 (12.6%)

[a]*Includes participants who reported "Prefer not to disclose" or had missing values*

© 2026 Moreland CJ et al. *JAMA Network Open*

[b]*Under-represented in medicine (URiM) includes participants who self-reported at least one of the following race/ethnic identities: Latinx/Latino/Hispanic; Native American/American Indian/Indigenous or Alaskan Native; Black/African American/Afro-Caribbean; Native Hawaiian/Pacific Islander.*
[c]*Other includes participants who self-reported as Middle East and North African (MENA), or non-URIM multiracial (e.g., MENA/White, White/Asian, MENA/Asian).*
[d]*Participants were allowed to select more than one type of disability. Percentages may sum to greater than 100%.*

© 2026 Moreland CJ et al. *JAMA Network Open*
