# Supplementary material for: Disability Accommodation Access and Requests in US Internal Medicine Residents With Disabilities
Source: JAMA Netw Open. 2026 Mar 30;9(3):e263392. doi: 10.1001/jamanetworkopen.2026.3392 (PMC13036577; doi:10.1001/jamanetworkopen.2026.3392)
Supplement: Supplement 2. — Data Sharing Statement [file jamanetwopen-e263392-s002.pdf]

Data Sharing Statement

Moreland. Disability Accommodation Access and Requests in US Internal Medicine Residents With Disabilities. *JAMA Netw Open*. Published March 30, 2026. doi:10.1001/jamanetworkopen.2026.3392

Data

Data available: No
